# Computed tomography image quality with high-flow contrast via high-pressure central venous catheter in critically ill patients

**DOI:** 10.1186/s13054-025-05673-4

**Published:** 2025-10-14

**Authors:** Emmanuelle Gentil, Quentin de Roux, Solène Ribot, Lucien Lapeyre, Victor PalombI, Alain Luciani, Christophe Quesnel, Vania Tacher, Nicolas Mongardon

**Affiliations:** 1 Service d’anesthésie-réanimation et médecine péri-opératoire, Assistance Publique-Hôpitaux de Paris, Hôpitaux Universitaires Henri Mondor, Créteil, F-94010 France; 2 Service d’imagerie médicale, Assistance Publique-Hôpitaux de Paris, Hôpitaux Universitaires Henri Mondor, Créteil, F-94010 France; 3 Univ Paris Est Créteil, INSERM, IMRB, Créteil, F-94010 France; 4 Ecole Nationale Vétérinaire d’Alfort, IMRB, Maisons-Alfort, F-94700 France; 5 Faculté de Santé, Université Paris Est Créteil, Créteil, F-94010 France; 6 GRCT OPTIMA, Université Paris Est Créteil, Créteil, F-94010 France

Dear Editor,

Contrast-enhanced computed tomography (CECT) is a key diagnostic procedure in critically ill patients, in whom contrast media (CM) is injected most of the time through central venous catheter (CVC). The quality of contrast enhancement relies on patient-dependent (cardiac output, weight and size) or patient-independent (technology of CT-scan, type of catheter, site of injection, CM volume, and injection rate) factors [1]. Injection rate is a crucial parameter of contrast enhancement, but is limited by the intrinsic quality of the venous access, with risks of displacement, ballooning, rupture and contrast extravasation in case of high-pressure (HP) or high-flow rate [2]. While standard CVC allow injection rate up to 5 mL/sec, manufacturers do not recommend to use them for pressure injection [3]. Thus, CVCs allowing higher pressures and flow rates have been recently designed [4]. This could improve the image quality of CECT, or reduce CM volume for similar quality, thereby reducing the risk of contrast-induced acute kidney injury and minimizing environmental impact. However, the interest of CM injection at high-flow through HP CVC has never been investigated. Here we report on the objective image quality of CECT images obtained with CM injected at high-flow rate through HP CVC versus standard flow rates through standard CVC. All critically ill adult patients with superior vena cava CVC requiring thoracic and/or abdominal CECT were analyzed. Before September 2023 (retrospective/before period), standard CVC were used (references: CV-12703, CV-15703, CV-12854, CV-15854, Arrow^®^/Teleflex^®^). After September 2023 (prospective/after period), HP CVC were used (references: EU-42854-HPS, EU-45854-HPS, Arrow^®^/Teleflex^®^). Patients on ECMO or requiring isolated head CT-scan were excluded. Except flow rate, all CT-scans with CM injection were performed according to the same protocol. CM injection was performed with the device Bracco Injeneering SA^®^ (650197 CT Expres™ - Control Panel). Monoergenetic CT acquisitions were performed with CT device (Revolution CT Apex Elite, General Electric Medical Systems^®^). The injection sequence was 10 mL of isotonic saline, CM injection with volume set at 1 mL/kg, and purge of 20 mL of isotonic saline at the same flow rate. In the standard CVC group, the flow rate was set of 3.5 mL/s, with injection through the 16-gauge distal lumen; in the HP CVC group, the flow rate was set at 10 mL/s, with injection through the dedicated 14-gauge medial lumen. Image quality was assessed based on combination of aortic and liver Signal-to-Noise Ratio (SNR) and Contrast-to-Noise Ratio (CNR) at arterial and portal time at the level of coeliac trunk in aorta and in liver, with blind analysis by a senior radiologist. Briefly, the SNR quantifies the strength of a signal relative to the background noise. It is calculated by dividing the mean signal intensity by the standard deviation of the noise in a round region of interest of 3cm^2^. A higher SNR indicates a clearer and more distinct signal [5]. The CNR measures the contrast between the signal of interest and the background, relative to the noise. It is determined by dividing the difference in signal intensity between a 3cm^2^ round region of interest and a reference region, such as muscle, by the standard deviation of the noise. In this study, noise was air. A higher CNR indicates a better contrast [5]. During the two periods, a total of 75 patients were included: 56 patients had 67 CECT with standard CVC and 19 patients had 31 CECT with HP CVC. Table 1 displays main characteristics of patients, factors influencing cardiac output, and CECT. There was no clinically relevant difference between the groups. Flow rate was significantly higher in the HP CVC group, with a median flow rate of 7.7 mL/s [7- 8.3] compared to 3.5 mL/s in the standard CVC group (*p* < 0.001). Volume of CM or iodine dose normalized to body weight were similar. However, there were no significant differences in aortic and liver SNR and CNR measurements during the arterial and portal phases. This preliminary report, as the first series evaluating the image quality of CECT with high-flow CM injection via HP CVCs to enhance, deserves some limitations. Firstly, the theoretical flow rate of 10 ml/s was not achieved, with an effective median flow rate of 7.7 mL/s. Possible explanations are limits of calibration of the power injector, or changes in the CM viscosity, which depends on the ambient room temperature which could have varied over time. Secondly, we estimated a flow rate of 3.5 mL/s in the standard CVC group because this information could not be retrieved retrospectively. However, the exact flow rates remain unknown in both groups. Finally, the delay between CM injection and image acquisition was unchanged whatever the CM injection flow rate, but acquisition timing might be adjusted accordingly [1]. To conclude, in this series focusing on critically ill patients, the use of HP CVC allowed high-flow injection of CM without any adverse events. However, this strategy was not associated with improved objective image quality. Further studies are needed in specific populations, particularly those with cardiac output modifications in whom CECT quality is often reduced (such as patients on ECMO, with sickle cell disease, or pregnancy), or indications (research of pulmonary embolism or CT coronary angiogram).

**Table 1:** Baseline characteristics

	CECT obtained with standard CVC (*n* = 67)	CECT obtained with high-pressure CVC (*n* = 31)	*p*
***Baseline characteristics of the population***			
**Number of patients**	56	19	/
**Age (years)**	67 [61–72]	56 [61–75]	0.80
**Male sex**	45 (80)	15 (79)	1.00
**SAPS II**	49 [36–63]	66 [37–68]	0.84
**BMI(kg/m** ^**2**^ **)**	26.9 [23.1–29.9]	22.9 [22.3–33]	0.94
***Factors influencing contrast media distribution at the time injection***			
**Invasive mechanical ventilation**	46 (69)	21 (68)	1.00
**Hemoglobin level (g/dL)**	9.1 [8.1–11]	8.4 [8-10.2]	0.06
**Norepinephrine: ** **- Infusion** **- Dose (µg/kg/min)**	37 (55) 0.04 [0-0.39]	11 (35) 0 [0-0.15]	0.08 0.12
**Dobutamine:** **- Infusion ** **- Dose (µg/kg/min)**	2 (3) 0 [0–0]	0 (0) 0 [0–0]	1.00 0.34
**Site of CVC injection** **- Right jugular vein** **- Left jugular vein** **- Right subclavian vein** **- Left subclavian vein**	40 (60) 26 (39) 1 (1) 0 (0)	17 (55) 10 (32) 0 (0) 4 (13)	0.67 0.65 1 0.01
***Modalities of contrast media injection***			
**Contrast media:** **- volume (mL)** **- volume adjusted to body weight (mL/kg)**	90 [80–90] 1.1 [1.0-1.3]	85 [71–90] 1.0 [0.9–1.1]	0.04 0.10
**Type of contrast media used** **- Iomeron 350 ** **- Iomeron 400** **- Xenetix 350**	57 (87) 7 (10) 2 (3)	28 (90) 2 (7) 1 (3)	0.74 0.71 1
**Iodine dose (g iode/kg)**	0.4 [0.35–0.44]	0.35 [0.31–0.39]	0.07
**Flow rate injection (mL/sec)**	3.5	7.7 [7-8.3]	< 0.001
***Assessment of image quality***			
**Signal-to-Noise Ratio** **- Aortic arterial phase** **- Liver arterial phase** **- Aortic portal phase** **- Liver portal phase**	23.9 [11.8–38.8] 3.9 [2.1–7.2] 9.3 [4.8–15.8] 6.3 [3.5–11.0]	25.5 [12.2–66.7] 4.5 [1.8–16.2] 7.3 [3.3–21.3] 4.5 [2.8–14.9]	0.42 0.52 0.60 0.70
**Contrast-to-Noise Ratio** **- Aortic arterial phase** **- Liver arterial phase** **- Aortic portal phase** **- Liver portal phase**	18.7 [9.6–35.2] 0.9 [−0.1-2.1] 4.6 [2.2–9.9] 2.3 [1.1–5.2]	18.3 [8.2–61.0] 1.2 [0.3-5.0] 3.7 [2.2–6.7] 2.0 [1.2–4.8]	0.59 0.24 0.78 0.91

Table Data are expressed as median [interquartile 25–75] or number (percentage), as appropriate

BMI: body mass index; SAPS 2:Simplified Acute Physiology Score 2

## Data Availability

Data are available on reasonable request to the corresponding author.

## Abbreviations

CECT: Contrast-enhanced computed tomography
CM: Contrast media
CNR: Contrast-to-noise ratio
CT: Computed tomography
CVC: Central venous catheter
ECMO: ExtraCorporeal Membrane Oxygenation
HP: High-pressure
ICU: Intensive care unit
SNR: Signal-to-noise ratio
